# Supplementary material for: Cancer-Associated-Fibroblast-Mediated Paracrine and Autocrine SDF-1/CXCR4 Signaling Promotes Stemness and Aggressiveness of Colorectal Cancers
Source: Cells. 2024 Aug 12;13(16):1334. doi: 10.3390/cells13161334 (PMC11352219; doi:10.3390/cells13161334)

Supporting information

Table S1. pMSCV-SDF-1 coding sequence

----

pMSCV-Coding sequence

ATGAAGCCTAAAATGAAGTATTCAACCAACAAAATTTCCACAGCAAAGTGGAAGAAC
ACAGCAAGCAAAGCCTTGTGTTTCAAGCTGGGAAAATCCCAACAGAAGGCCAAAGA
AGTTTGCCCCATGTACTTTATGAAGCTCCGCTCTGGCCTTATGATAAAAAAGGAGGCCT
GTTACTTTAGGAGAGAAACCACCAAAAGGCCTTCACTGAAAACAGGTAGAAAGCAC
AAAAGACATCTGGTACTCGCTGCCTGTCAACAGCAGTCTACTGTGGAGTGCTTTGCCT
TTGGTATATCAGGGGTCCAGAAATATACTAGAGCACTTCATGATTCAAGTATCACAGGA
ATTTCACCTATTACAGAGTATCTTGCTTCTCTAAGCACATACAATGATCAATCCATTACT
TTTGCTTTGGAGGATGAAAGTTATGAGATATATGTTGAAGACTTGAAAAAAGATGAAA
AGAAAGATAAGGTGTTACTGAGTTACTATGAGTCTCAACACCCCTCAAATGAATCAGG
TGACGGTGTTGATGGTAAGATGTTAATGGTAACCCTGAGTCCTACAAAAGACTTCTGG
TTGCATGCCAACAACAAGGAACACTCTGTGGAGCTCCATAAGTGTGAAAAACCACTG
CCAGACCAGGCCTTCTTTGTCCTTCATAATATGCACTCCAACTGTGTTTCATTTGAATGC
AAGACTGATCCTGGAGTGTTTATAGGTGTAAAGGATAATCATCTTGCTCTGATTAAAGT
AGACTCTTCTGAGAATTTGTGTACTGAAAATATCTTGTTTAAGCTCTCTGAAACTTAG

Human SDF-1Protein sequence: 93 aa

MNAKVVVVLVLVLTALCLSDGKPVSLSYRCPCRFFESHVARANVKHLKILNTPNCALQIVA
RLKNNNRQVCIDPKLKWIQEYLEKALNKRFKM

Table S2. List of antibodies used in this study.

Protein	Dilution	Assay	Origin
SDF-1	1:1000/1:100	WB/IHC	ab18919, abcam
CXCR4	1:1000/1:200	WB/IHC	ab124824; abcam
α-Smooth muscle actin	1:1000/1:200	WB/IF	ab5694; abcam
CD10	5 μl/1x10^6	Flow	GTX78263, Genetex
GPR77	5 μl/1x10^6	Flow	MAB10254, R&D systems
Twist1	1:1000	WB	GTX127310, GeneTex
Snail	1:1000	WB	GTX125918, GeneTex
E cadherin	1:1000	WB	GTX1004431 GeneTex
Vimentin	1:2000/1:300	WB/IF	GTX100619i GeneTex
CD-44	1:1000/1:300	WB/IHC	MAS-13890, Thenno Scienticfic
CD-133	1:1000	WB	ab19898, abcam
OCT-4	1:1000/1:200	WB/IHC	D73G4, Cell Signaling Technology
Nanog	1:1000/1:200	WB/IHC	D6D9, Cell Signaling Technology
ABCG-2	1:1000	WB	sc-55510, Santa Cruz Biotechnology
MDR-1	1:1000	WB	D3U1W, Cell Signaling Technology
PE-CD44	5 μl/1x10^6	Flow	Cat. No. 338808; BioLegend
PE-CD133	5 μl/1x10^6	Flow	Cat. No. 393903; BioLegend
PE-Mouse IgG1, κisotype	5 μl/1x10^6	Flow	Cat. No. 400113; BioLegend
a-Tubulin	1:5000	WB	GTX628802, GeneTex

Table S3. List of primers used in this study.

Name	Sequence
SDF-1	F: 5′- TTGACCCGAAGCTAAAGTGG
	R: 5′- TGGGCTCCTACTGTAAGGGTT
CXCR4	F: 5′-cagagacagcagagcacacaagc
	R: 5′-gagatggttccttccggtggt
FAP	F: 5′-GGAAGTGCCTGTTCCAGCAATGG
	R: 5′-TGTCTGCCAGTCTTCCCTGAAG
a-SMA	F: 5′- CTATGCCTCTGGACGCACAACT
	R: 5′- CAGATCCAGACGCATGATGGCA
Vimentin	F: 5′- CTT CAG AGA GAG GAA GCC GA
	R: 5′- ATT CCA CTT TGC GTT CAA GG
GAPDH	F: 5′-TCCACTGGCGTCTTCACC
	R: 5′-GGCAGAGATGATGACCCTTTT

Table S4. Sensitivity and Specificity of IHC in this study.

	**Clinical Outcome Positive**	**Clinical Outcome Negative**
**Test Positive**	TP = 23	FP = 7
**Test Negative**	FN = 2	TN = 8

$$\text{Sensitivity}=\frac{23}{23+2}=0.92 \text{ or } 92\%$$

$$\text{Specificity}=\frac{8}{7+8}=0.53 \text{ or } 53\%$$

Figure S1. The induction of HT-29 and HCT-116 SDF-1 secretion by CAF-conditioned medium under enzyme-linked immunosorbent assays (ELISA).

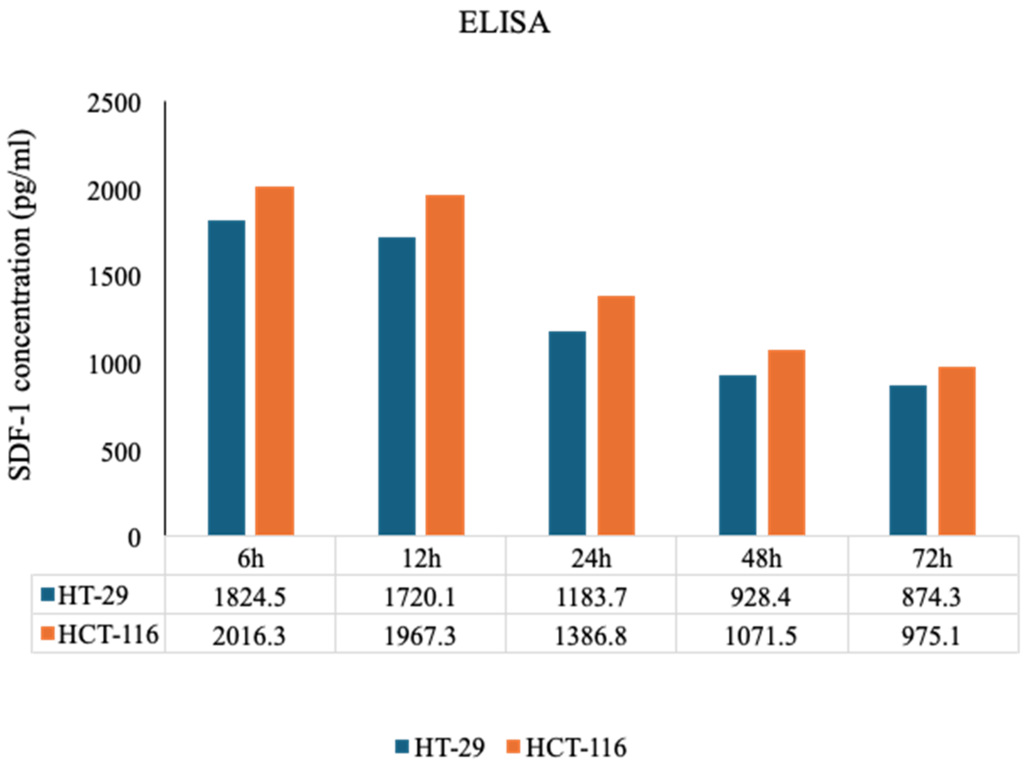

Figure S2. The utility of SDF1 and CXCR4 score as violin plots represents the distribution and probability density staining correlates the histopathological CRC aggressiveness.

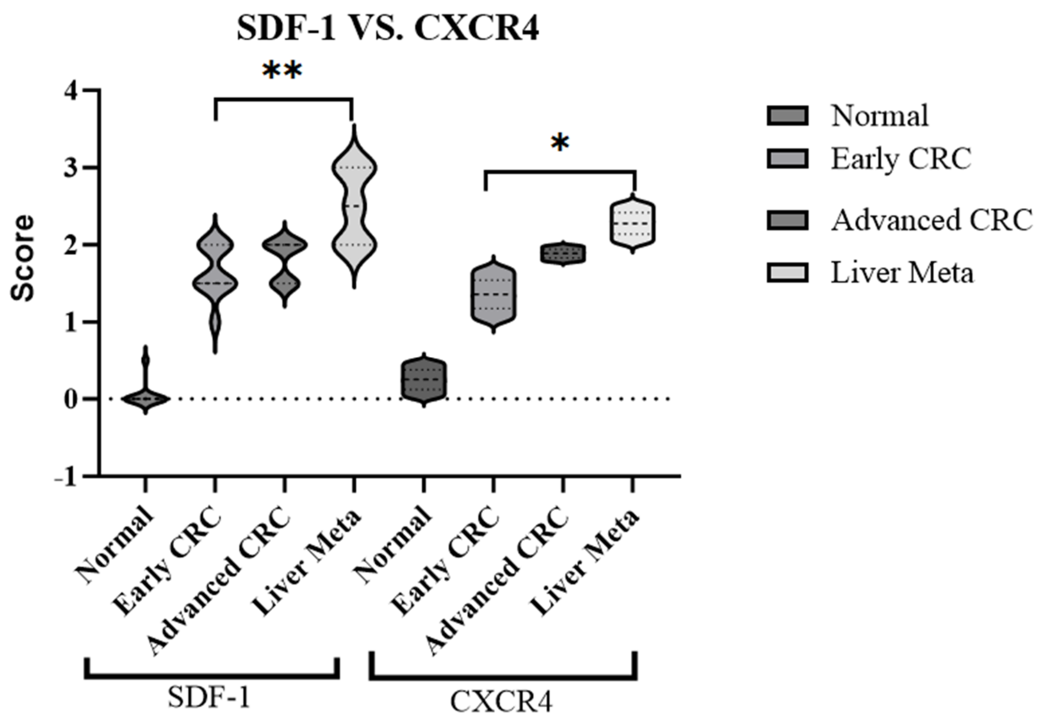

Supplement: Supplementary file 1 [file cells-13-01334-s001.zip › cells-3051538-supplementary.pdf]
